# Molecular Mechanisms Underlying the Effects of Statins in the Central Nervous System

**DOI:** 10.3390/ijms151120607

**Published:** 2014-11-10

**Authors:** Amelia J. McFarland, Shailendra Anoopkumar-Dukie, Devinder S. Arora, Gary D. Grant, Catherine M. McDermott, Anthony V. Perkins, Andrew K. Davey

**Affiliations:** 1 School of Pharmacy, Griffith University, Queensland 4222, Australia; E-Mails: a.mcfarland@griffith.edu.au (A.J.M.); s.dukie@griffith.edu.au (S.A.-D.); d.arora@griffith.edu.au (D.S.A.); g.grant@griffith.edu.au (G.D.G.); 2 Griffith Health Institute, Griffith University, Queensland 4222, Australia; E-Mail: a.perkins@griffith.edu.au; 3 Department of Biomedical Science, Bond University, Queensland 4226, Australia; E-Mail: camcderm@bond.edu.au; 4 School of Medical Sciences, Griffith University, Queensland, 4222, Australia

**Keywords:** statin, cognition, central nervous system (CNS), neurotoxicity, neuroprotection

## Abstract

3-Hydroxy-3-methylglutaryl coenzyme A reductase inhibitors, commonly referred to as statins, are widely used in the treatment of dyslipidaemia, in addition to providing primary and secondary prevention against cardiovascular disease and stroke. Statins’ effects on the central nervous system (CNS), particularly on cognition and neurological disorders such as stroke and multiple sclerosis, have received increasing attention in recent years, both within the scientific community and in the media. Current understanding of statins’ effects is limited by a lack of mechanism-based studies, as well as the assumption that all statins have the same pharmacological effect in the central nervous system. This review aims to provide an updated discussion on the molecular mechanisms contributing to statins’ possible effects on cognitive function, neurodegenerative disease, and various neurological disorders such as stroke, epilepsy, depression and CNS cancers. Additionally, the pharmacokinetic differences between statins and how these may result in statin-specific neurological effects are also discussed.

## 1. Introduction

3-Hydroxy-3-methylglutaryl coenzyme A (HMG-CoA) reductase inhibitors, more commonly referred to as statins, are a class of cholesterol-lowering agents used for the treatment of dyslipidaemia and reduction of atherosclerotic cardiovascular disease risk. Their broad and potent effects on the lipid profile, in conjunction with cholesterol-independent (pleiotropic) cardioprotective effects, have resulted in statins being amongst the most highly prescribed medications worldwide. In spite of high patient tolerability, concerns over the neurological effects of statins have emerged in recent years. Although individual case reports form the basis of these concerns, larger studies and trials have yielded different conclusions, with negligible or in some cases beneficial actions being reported. Whilst numerous clinical studies have sought to determine statins’ therapeutic potential in various central nervous system (CNS) disorders, including dementia, multiple sclerosis (MS), epilepsy, depression and stroke, there is still a lack of understanding surrounding the mechanisms of statins’ neurological effects. As such, unlike recent reviews and meta-analyses which explore the risks associated with statin use and the development of various neurological conditions (for these see [1,2,3,4]), this review specifically focuses on the molecular mechanisms of statins in the CNS, how pharmacokinetic differences may influence statin action, and subsequent differences in effect between statin compounds.

## 2. Pharmacology

### 2.1. Mechanism of Action

**Figure 1:**
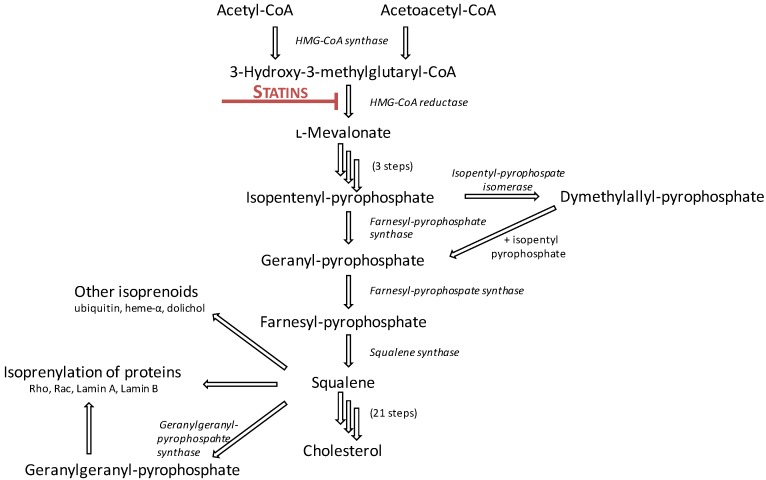
Statins inhibit the conversion of 3-hydroxy-3-methylglutaryl coenzyme A (HMG-CoA) to l-mevalonate, the rate-limiting step of the cholesterol synthesis pathway. Adapted from [10].

Statins’ primary mechanism of action is through the competitive, reversible inhibition of HMG-CoA reductase, the rate-limiting step in cholesterol biosynthesis. HMG-CoA reductase catalyses the conversion of HMG-CoA to l-mevalonate and coenzyme A via a four-electron reductive deacetylation (Figure 1). The pharmacophore of all statins bears resemblance to the endogenous HMG-CoA moiety (Table 1); it competitively binds to the catalytic domain of HMG-CoA reductase, causing steric hindrance and preventing HMG-CoA from accessing the active site [5,6].

Through inhibition of HMG-CoA reductase, statins ultimately prevent the endogenous production of cholesterol. Additionally, the resultant reduction in cholesterol concentration within hepatocytes triggers up-regulation of low-density lipoprotein (LDL)-receptor expression, which promotes the uptake of LDL and LDL-precursors from systemic circulation [7]. Consequently, a significant proportion of statins’ cholesterol-lowering is a result of the indirect increase in LDL clearance from plasma, as opposed to simply reduced cholesterol biosynthesis. Secondary mechanisms of statin-induced lipoprotein reduction include inhibition of hepatic synthesis of apolipoprotein B100, and the reduced synthesis and secretion of triglyceride-rich lipoproteins [8,9].

Overall, the effect on the lipid profile is consistent between statins, with reductions in total cholesterol, LDL, and triglycerides, and an increase high-density lipoprotein. Despite having the same mechanism of action and comparative effects on cholesterol profiles, statins can still be subdivided into one of two categories: type I, fungal-derived statins (lovastatin, pravastatin, simvastatin); or type II, synthetically-derived statins (fluvastatin, cerivastatin, atorvastatin, rosuvastatin, pitavastatin). Type I statins maintain close structural homology to mevastatin, the first statin to be developed, maintaining the lactone/open acid moiety in addition to the substituted decalin ring skeleton (Table 1). Although type II statins maintain the HMG-CoA-like lactone moiety for binding, these compounds are fully synthetic inhibitors of HMG-CoA reductase and exhibit highly varied pharmacokinetic properties, including differences in metabolism, excretion, half-lives, bioavailability, dosing times and lipophilicity.

### 2.2. Pharmacokinetics

Upon oral administration all statins are well absorbed from the intestine, though they undergo extensive first-pass metabolism within the liver, which reduces systemic bioavailability to 5%–50% [11,12]. Most statins are administered as β-hydroxy-acids except for lovastatin and simvastatin, which are pro-drugs and require hepatic metabolism to their active β-hydroxy-acid state. Within the systemic circulation statins can bind variably to albumin, and also differ substantially with respect to half-life and volume of distribution [5,12]. The predominant metabolism route of most statins is via cytochrome P450 (CYP), with atorvastatin, lovastatin and simvastatin metabolised through isoform CYP3A4, and fluvastatin metabolised through isoform CYP2C9 [5,12,13]. In contrast, pravastatin is metabolised largely through sulfation, whilst up to 90% of rosuvastatin is removed via biliary excretion [5,12,13,14]. Differences in published reports surrounding the pleiotropic effects and adverse effect profiles between statins may be a direct result of their highly varied pharmacokinetic parameters.

**Table 1:** Pharmacokinetic (PK) characteristics of commonly prescribed statins, data summarised from [5,11,13,14,15,16,17,18,19,20,21,22,23]. Blue-coloured moiety in chemical structures indicates the pharmacophore. * Logarithms of octanol–water distribution coefficients (log *D*) are presented at pH 7.0 for rosuvastatin and pH 7.4 for all other drugs.

PK Parameter	Atorvastatin	Fluvastatin	Lovastatin	Pitavastatin	Pravastatin	Rosuvastatin	Simvastatin
**Molecular Structure**	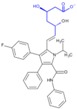	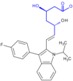	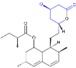	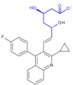	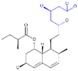	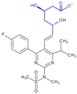	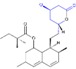
Statin Type	II	II	I	II	I	II	I
Dosing Time	Any time of day	Bedtime	With food morning & night	Any time of day	Bedtime	Any time of day	Evening
Prodrug	No	No	Yes	No	No	No	Yes
Bioavailability	12%	9%–50%	5%	51%	18%	20%	<5%
Half-Life	14 h	2.3 h	3 h	12 h	1.3–2.7 h	19 h	3 h
Volume of Distribution	381 L	330 L	(not available)	148 L	35 L	134 L	(not available)
Log D *	1.53	1.75	3.91 (lactone)/1.51 (acid)	1.50	−0.47	−0.25 to −0.50	4.40 (lactone)/1.80 (acid)
Lipophilicity	Lipophilic	Lipophilic	Lipophilic	Lipophilic	Hydrophilic	Hydrophilic	Lipophilic
Active Metabolites	Yes	No	Yes	Yes (minimal)	Yes (minimal)	Yes (minimal)	Yes
CYP Substrate	3A4	2C9	3A4	2C8, limited 2C9 *mostly glucuronidation*	Limited 3A4 *mostly sulfation*	Limited 2C9 *mostly excreted unchanged*	3A4
Effects on p-Glycoprotein	Substrate and inhibitor	No significant inhibition	Substrate and inhibitor	No significant inhibition	No significant inhibition	No significant inhibition	Substrate and inhibitor
OATP Transporters	1B1, 2B1	1B1, 1B3, 2B1	1B1	1A2, 1B1, 1B3	1B1, 1B3, 2B1	1A2, 1B1, 1B3, 2B1	1B1
Protein Binding	Very high (98%)	Very high (98%)	Very high (95%)	Very high (96%)	Moderate (50%)	High (90%)	Very high (95%)
Excretion (Renal)	<2%	6%	10%	2%	20%	10%	13%
Excretion (Faecal)	>98%	93%	83%	79%	70%	90%	60%

## 3. Statins in the Central Nervous System (CNS)

### 3.1. Effects on Brain Cholesterol

For the most part, cholesterol in the adult brain is largely metabolically inert, with an estimated 0.02% undergoing turnover daily [24]. The most significant period of high cholesterol synthesis in the CNS occurs during active myelination, which occurs in early neural development, through the action of oligodendrocytes (ODs) [25]. The rate of cholesterol synthesis decreases significantly after myelination has been completed, however it does still continue at a low basal level in the mature adult brain. This occurs primarily through *de novo* cholesterol synthesis by astrocytes, although neuronal *de novo* synthesis and reutilisation of free cholesterol following neuronal death also contributes [26,27].

Whilst the effect of statins on the peripheral pool of cholesterol is well-established, statins’ effects on CNS cholesterol are less clear. The CNS does not rely largely on cholesterol from systemic circulation due to limited metabolic turnover during adulthood and the brain’s inherent capacity to synthesise its own cholesterol [25]. As such, reductions in plasma cholesterol concentration following statin treatment are unlikely to cause acute disruption in CNS cholesterol homeostasis [28,29]. Unlike cholesterol in plasma which has a half-life of only a few days [24], brain cholesterol has been associated with a half-life of from 6 months to 5 years [30,31]. Thus, chronic statin therapy may be required before significant effects on CNS cholesterol are seen, with reductions in CNS cholesterol possible either directly through direct HMG-CoA reductase inhibition, or indirectly via a “sink effect” [32].

24(*S*)-Hydroxycholesterol has been used in many studies as an indicator of brain cholesterol turnover, as it is the by-product of cholesterol metabolism through brain-selective cholesterol 24-hydroxylase (CYP46A1) and is capable of passing through the blood-brain barrier (BBB) for detection in systemic circulation. Following chronic statin administration, numerous studies have shown reductions in plasma and cerebrospinal fluid (CSF) concentrations of 24(*S*)-hydroxycholesterol [33,34,35,36,37,38,39]. This is in-line with a reduced elimination of cholesterol in the brain as a result of prolonged statin treatment, and suggests statins may indeed affect cholesterol homeostasis in the brain. Thus, considering the low turnover rates of cholesterol within the CNS, is it possible that chronic statin administration is required for any changes in brain cholesterol levels to be observed.

### 3.2. CNS Entry

The key question of whether statin compounds differ in their ability to permeate the CNS often emerges when considering neurological effects of statins. Whilst lipophilic statins (atorvastatin, lovastatin, fluvastatin, pitavastatin, simvastatin) are capable of crossing the BBB passively, both *in vitro* and *in vivo* studies suggest that hydrophilic statins are also able to enter the neuroparenchyma [28,40,41]. Pravastatin has been shown to induce gene expression changes within the mouse brain [40] and has also been detected in human CSF [41] which, considering its poor lipid solubility, raises the question of whether active transporters within the BBB facilitate its entry. All statins, including rosuvastatin and pravastatin, are known substrates for organic anion transporting polypeptides (OATP; Table 1), of which OATP1A2 and OATP1C1 are known to be expressed in the brain [22,42]. While it is possible that OATP-mediated influx may be a mechanism for hydrophilic statin entry, there have been no studies to date which explore the selectivity of these statins for the CNS-expressed OATP subtypes. Additionally, the presence of monocarboxylic acid transporters at the BBB may represent an alternative mechanism of CNS entry, with pravastatin shown to have affinity for monocarboxylic acid transporters in intestinal epithelial barriers [43], although studies specific to the CNS are again lacking.

Regardless of specific transporters, statins are likely to accumulate at differing rates and concentrations within the CNS based upon their differing lipid solubilities alone. When also considering their vast structural differences, their propensity for carrier-mediated uptake into the CNS may also vary between compounds. The possible variations in CNS entry, efflux and indeed potency between statins highlight the need for these drugs to be considered individually with respect to their CNS actions. Until such time that quantification of CNS uptake and efflux for each statin can be achieved, the assumption that statins’ effects within the CNS are equivalent and thus broadly applicable across the whole class should be reconsidered.

## 4. Statins and Cognition

Despite a plethora of literature available, the effects of statins on cognitive function remain controversial [2,44,45,46,47,48,49]. Whilst increasing epidemiological evidence suggests a role for statins in neurodegenerative conditions including vascular dementia, Alzheimer’s disease (AD) and Parkinson’s disease (PD), there are also several large studies in addition to a number of case reports which contradict these findings (see summary of mechanisms and evidence in Table 2). Given the previously discussed pharmacokinetic differences between statins in the CNS, it is plausible that the differences between studies thus far may be explained by different statin molecules exerting varying degrees of cognitive effect, however this remains speculative. The lack of information surrounding the molecular mechanism of action of statins in the CNS further compounds this uncertainty.

**Table 2:** Summary of statins’ effects on cognition and neurocognitive disorders.

Disorder	Possible Statin-Induced Mechanisms	Strength of Evidence	Overall Consensus
General cognition	↓ FPP and/or GGPP; modulation of adult neurogenesis; ↑ expression of neural growth factors.	Limited *in vitro* and *in vivo* studies. Conflicting evidence from epidemiological studies and randomised controlled trials. Case reports of negative effects on cognition. Recent meta-analyses suggest long term statin use may reduce incident dementia.	Long-term statin treatment appears to be beneficial for cognitive function. Whether statins can cause acute cognitive disruption as a rare adverse effect is unclear due to lack of causal evidence from case reports. Identification of underlying mechanisms *in vitro* or *in vivo* is difficult due to the subjective nature of acute cognition changes.
Alzheimer’s disease	↓ FPP and/or GGPP; ↓ APP production; ↓ ROCK activity; ↓ amyloid-β production; ↑ amyloid-β degradation; ↓ neuroinflammation; ↓ ROS.	Numerous *in vitro* and *in vivo* studies, however some data appears model-dependent so requires careful interpretation. Several randomised controlled trials, and multiple systematic reviews and meta-analyses have been conducted.	Studies suggest statins, if started before old age and without cognitive dysfunction at baseline, may reduce incidence of AD. It is likely different statins have different capacities for inducing this effect.
Parkinson’s disease	↓ ROS; ↓ nitric oxide; ↓ lipid peroxidation; ↓ neuroinflammation; ↓ NF-κB activity; ↓ neuronal loss.	Numerous *in vitro* and *in vivo* studies, however data from prospective studies or clinical trials is lacking.	Data from cell and animal models is encouraging, however further well-designed prospective studies are needed to evaluate statins’ clinical application in PD.
Multiple sclerosis	Altered Th1/Th2 ratio; ↓ neuroinflammation; ↑ remyelination-associated genes; ↑/↓ differentiation from OPC to OD; ↓ ROCK activity; modulation of NF-κB activity.	Numerous *in vitro* and *in vivo* studies, however results from these are highly conflicting. Simvastatin has recently completed phase II testing as a treatment for MS.	Vast discrepancies between models limits our understanding of the mechanisms of statins in MS. It appears likely that modulation of neuroinflammation and/or T cell immunity is involved. Further studies needed to determine if benefit is seen with statins other than simvastatin in MS.
Neurofibromatosis Type I	↓ Ras activity; rescue long-term potentiation deficit.	Limited *in vitro* and *in vivo* data. Conflicting data from randomised controlled trials.	Further cell and animal studies are recommended to better understand possible clinical application in NF-1 before any further trials in children with the disorder are conducted.

### 4.1. Cognitive Function

The effects of statins on cognitive function have received increasing, and arguably disproportionate, attention in recent years. Data from clinical trials thus far has been inconsistent, not only in terms of results, but also analytical methods, population characteristics, existence of baseline cognitive impairments, statin(s) studied, and cognitive endpoints employed. Despite these differences, the majority of studies support a role for protection against cognitive impairment and dementia in patients without baseline cognitive dysfunction following long-term statin use [2,47,49,50,51]. A recent meta-analysis found that in long-term cognition studies, incident dementia was reduced in statin-treated patients (hazard ratio, 0.71; 95% confidence interval, 0.61–0.82) [2].

A number of mechanisms have been implicated in statin-induced protection against cognitive impairment, including both cholesterol-dependent and -independent mechanisms. Increased LDL levels and total cholesterol have both been independently associated with cognitive impairment, thus the lowering of these lipoprotein levels, through statin treatment or other pharmacological/dietary means, has been suggested as a strategy for preventing cognitive impairment [52,53]. Despite this apparent disease link, statins have not only been implicated in cholesterol-associated reductions in cognitive impairment, but have also been found to reduce the odds of cognitive impairment independent of lipid levels [54].

Although HMG-CoA reductase is the rate-limiting step of cholesterol biosynthesis in humans, it is only the second step of a 28-step process (see Figure 1). Consequently, statin treatment also prevents the production of a number of intermediary molecules, including isoprenoid products such as farnesylpyrophosphate (FPP) and geranylgeranylpyrophosphate (GGPP). It has been suggested that much of the cholesterol-independent actions of statins may be attributable to the inhibition of these isoprenoids, including effects on cognitive function. The inhibition of farnesylation by simvastatin has been associated with the enhancement of long-term potentiation between neurons in mice [55]. This study also found that the protective effect of statin treatment was abolished following replenishment of FPP, but not GGPP. Paradoxically, it has been suggested in other studies that the constant production of GGPP, but not FPP or cholesterol, is required for neurite outgrowth and maintenance, long-term potentiation and learning [56,57], possibly suggested differing neuroprotective effects associated with these two isoprenoid intermediates. Given the different roles each of these compounds has, known differences in FPP/GGPP ratios across various brain regions may subsequently result in different local statin-induced effects within these regions. The mechanisms underlying the differential distribution of FPP and GGPP across the brain, and the interplay this has with statin effect, are not known.

Another possible cellular mechanism which may underlie the possible beneficial cognitive effect of statins is the alteration of adult neurogenesis. It is hypothesized that suppression of adult neurogenesis may contribute to cognitive dysfunction and emotional symptoms in neurological and psychiatric disorders, with neuroinflammation shown to be an inhibitor of neurogenesis in the adult hippocampus [58,59]. Simvastatin has been shown to enhance neurogenesis in cultured adult neural progenitor cells, as well as in the dentate gyrus of adult mice through enhanced Wnt signalling [60]. In several models of traumatic brain injury (TBI), statins have shown promise in enhancing neurogenesis, and in some have been associated within reductions in injury-associated neurological sequelae, including reduced cognitive deficit. Both simvastatin and atorvastatin have been shown to enhance neurogenesis in the dentate gyrus following TBI in rats [61,62], which was associated with increased vascular endothelial growth factor (VEGF) and brain-derived neurotrophic factor (BDNF) expression [62], increased cellular proliferation and differentiation in the dentate gyrus [62], reduced delayed neuronal death in the hippocampus [61], and improved spatial learning [61,62].

Despite meta-analyses suggesting no adverse effect on cognition resulting from statin treatment in the short-term [2], case reports of impairment in the form of transient, reversible memory loss and confusion have been published [45]. The presentation of detrimental cognitive symptoms is highly varied, both in terms of the nature of impairment (memory loss, amnesia, mood changes), and duration of statin therapy before onset (from 2 days to several months). The prevalence of these adverse effects across published data from large scale clinical trials and epidemiological studies appears negligible [44], however inconsistency of reporting and the risk of bias should be acknowledged. The question of how and why this phenomenon occurs remains unanswered, largely due to the extremely rare nature of this effect and uncertainties over the causal nature of these observations. Due to the CNS’ self-reliance in terms of cholesterol production, and the low metabolic turnover of cholesterol within the brain, it would be unlikely that an acute disruption in cholesterol synthesis in either the peripheral or CNS pool would contribute to acute cognitive impairment. This leaves cholesterol-independent, or so-called pleiotropic mechanisms implicated in this rare potential adverse effect.

### 4.2. Alzheimer’s Disease

In addition to statins’ acute cognitive effects, much attention has been devoted to the impact of statins both in the prevention and treatment of neurodegenerative disorders, such as AD. AD is a chronic, irreversible form of dementia, characterised by progressive memory loss and cognitive decline. The histopathology of AD is characterised by tissue atrophy and gliosis, in addition to synaptic loss predominating in the frontal and temporal cortices [63]. In addition to these structural features, intracellular neurofibrillary tangles (composed of hyper-phosphorylated tau protein) and extracellular amyloid plaques (composed of amyloid-β) are also typically seen throughout the brain parenchyma.

The first reports identifying the potential therapeutic benefit from statins in AD were two independent observational studies, whereby statin use was associated with reductions in AD occurrence of up to 70% [48,64]. Since this time, a number of clinical trials have been published with conflicting data. The majority appear to support this initial finding, that statin treatment in patients without baseline cognitive impairment and before old age may have a beneficial role in protecting against the onset of AD [47,50,51,65]. Furthermore, studies suggest that statins are unlikely to provide neuroprotection against disease progression in patients with existing cognitive impairment at baseline, or if initiated in late old age [50,51]. Consistent with the previous suggestion that individual statins may contribute differently to neurocognitive effects, a cross-sectional study by Wolozin and colleagues found lovastatin and pravastatin, but not simvastatin, to be associated with a reduced risk of AD development [64]. Given that statins are known to reduce dyslipidaemia, a known contributing factor for AD risk, cholesterol-dependent effects in the peripheries cannot be discounted as a mechanism for statins’ effects in reducing AD incidence. However, studies which identified that statins reduced the risk of developing dementia in patients with physiologically normal lipid profiles suggest that pleiotropic effects of statins may also contribute to this observed effect [48,53]. Several animal models of AD have shown statins to exert neurocognitive benefits in the absence of changes in plasma or brain cholesterol content, further suggesting a cholesterol-independent mechanism of protection [66,67,68].

A lack of information as to the true pathophysiology of AD limits our understanding of statins’ role in AD development and progression. A variety of experimental approaches have been used across both *in vitro* and *in vivo* studies, which has resulted in a number of proposed mechanisms of action of statins in AD. As with studies broadly exploring cognitive impairment, the depletion of isoprenoid intermediates has again been implicated as a possible mechanism for statin-mediated neuroprotection from AD. A study by Eckert and colleagues identified that both FPP and GGPP levels are significantly elevated in grey and white matter of human AD patients, however cholesterol levels were not [69]. This same study found that simvastatin treatment in mice significantly reduced brain levels of FPP and GGPP levels, though the effects of other statins are yet to be quantified [69]. Whether elevated FPP or GGPP levels are contributors to or consequences of AD neuropathophysiology remains unclear.

Whilst FPP and GGPP appear to mediate some of the effects of statins, it is likely that the downstream small GTPase family of signalling molecules also play an important role. These molecules, including Ras, Rho, Rac, Rab and Rap, are involved in the prenylation process, whereby their interaction with proteins increases lipophilicity and facilitates interaction with cellular membranes. Depletion of FPP and GGPP through statin treatment, and subsequent inhibition of these small GTPase proteins, has been associated with both neuroprotective and neurotoxic effects in various cell and animal models. The modulation of Alzheimer amyloid-β precursor protein (APP) metabolism has been implicated as one possible mechanism of neuroprotection, with both *in vitro* and *in vivo* studies demonstrating statin-induced attenuation of cerebral amyloidosis and APP production [66,70,71]. It has been suggested that the inhibition of the Rho-associated coiled-coil kinase1/2 (ROCK) pathway by both simvastatin and atorvastatin is a possible mechanism for stimulated soluble APP (sAPP) shedding in mouse N2a.Swe neuroblastoma cells [70]. A similar study using the same cell line identified that simvastatin preferentially increase sAPPα over total sAPP, however had no effect on other cell lines including mouse primary neurons and human neuroglioma cells, suggested that this response may be unique to this cell line [72]. Based on results from this study which compared the effects of lovastatin and simvastatin on APP processing across a number of cell types from human and mice, it is likely that statin-induced effects on APP metabolism are cell type-dependent, thus specific *in vitro* data surrounding APP processing should be analysed cautiously [72].

Despite statins’ actions on APP metabolism remaining unclear, a number of studies have consistently demonstrated reduced amyloid-β peptide (Aβ) production induced by statin treatment. In rat primary cortical neurons, treatment with either pitavastatin or atorvastatin (0.2–2.5 µM) induced time- and concentration-dependent reductions in Aβ40 and Aβ42 production [73]. Exogenous supplementation with cholesterol in this study did not restore Aβ levels, suggesting cholesterol-independent mechanisms underlying this observation.

Due to the apparent clinical link between statin use and reduced incidence/severity of inflammatory-based CNS pathologies, including AD, the reduction of chronic neuroinflammation has been proposed by many as a key mechanism for statin-induced neuroprotection. In experimental models of AD, the reduced production of Aβ has been attributed to reduction of neuroinflammation, and cells involved in the neuroinflammatory response [72,74]. In rats, atorvastatin prevented Aβ-induced microglial activation, an early step in the neuroinflammatory response [75]. Simvastatin (1–25 µM) was found to reduce Aβ-induced production of interleukin (IL)-1β in THP-1 monocytes, and reduced Aβ-induced and lipopolysaccharide (LPS)-induced nitric oxide, inducible nitric oxide synthase (iNOS) and reactive oxygen species (ROS) production in BV-2 microglial cells [76]. The release of inflammatory mediators, including IL-1β, IL-6, tumour necrosis factor (TNF)-α, and reactive nitrogen species, are also reduced by statins in astrocyte and macrophage models of Aβ-induced neuroinflammation [76,77,78], with these effects found to be mediated through Rho inhibition in THP-1 monocytes [76]. In contrast to the neuroprotective effects of Rho inhibition in microglia and monocytes, in a model of early AD using primary rat hippocampal neurons, lovastatin-induced apoptosis and cell death (10–100 µM) was attributed to Rho-dependent pathways [79]. Mevastatin treatment (10 µM) in cultured rat hippocampal slices has also been found to increase microglial activation [80]. These differences perhaps suggest a dose-dependent, statin-dependent and/or model-dependent relationship between statin use and models of neuroinflammation associated with AD.

Consistent with the attenuation of neuroinflammation, atorvastatin-induced reductions in brain oxidative and nitrosative stress have also been noted in aged beagles following chronic treatment (80 mg/day for 14.5 months) [78]; similar observations have been noted in other studies using mice, whereby atorvastatin (10 mg/kg for 7 days) and simvastatin (20 mg/kg for 8 weeks) both decreased oxidative stress and inflammatory levels, though neither treatment coincided with protection against cognitive impairment [81,82]. Duration of therapy may be an important factor in the neuroprotective potential of statins, with both atorvastatin (30 mg/kg/day) and pitavastatin (3 mg/kg/day) only showing protective effects against senile plaque and phosphorylated tau-positive dystrophic neuritis after 10 months of treatment in APP transgenic mice [67]. Another noteworthy variable is age, with simvastatin (40 mg/kg/day, 3–6 months) shown to fully restore short- and long-term memory in adult (6-month), but not in aged (12-month) transgenic mice [83]. Thus, the inconsistencies between studies thus far may be attributable to differing effects between statins, dose-dependent toxicities, time-dependent effects, cell-dependent responses and/or species-dependent responses.

Other mechanisms which have been implicated in statin-induced AD attenuation include: increased microglial degradation of extracellular Aβ in mice through farnesylation-dependent increases in insulin-degrading enzyme secretion (lovastatin, 5 µM) [84]; γ-secretase relocation in lipid rafts (pitavastatin, 5 µM) [85]; enhanced APP-*C* terminal fragment trafficking from endosomes to lysosomes [71]; and, reduced senile plaques and phosphorylated tau-positive dystrophic neuritis (atorvastatin 30 mg/kg/day, 15 months; pitavastatin 3 mg/kg/day, 15 months) [67]. On the whole, it would appear that statins exert some form of protection against early events associated with AD development. The lack of understanding as to the true pathophysiology of AD limits the application of cell and animal models of statin-mediated neuroprotection to the true mechanism of statins’ apparent effects. Given that the majority of studies use a single statin as a representative of the class, differences between individual statins’ mechanisms or propensity for neuroprotection against AD remains unclear.

### 4.3. Parkinson’s Disease

PD is a progressive neurodegenerative disorder characterised by the presence of Lewy bodies (intracellular protein aggregates), the loss of dopaminergic neurons from the substantia nigra *pars compacta* in the midbrain, and associated clinical manifestations of dopamine deficiency (gait, tremor, rigidity and bradykinesia). It is the second most common chronic neurodegenerative disorder in adults over the age of 65 years [86]. Epidemiological evidence suggests that some statins may reduce the incidence of PD; Wolozin and colleagues identified that simvastatin treatment was associated with significantly reduced incidence of PD in patients aged over 65 years, however neither atorvastatin nor lovastatin showed significant effects [49]. Compared with discontinuation of statins, continuation of lipophilic statin use has been associated with a reduced risk of PD, particularly in the elderly [87]. In patients with existing PD, however, 10 day treatment of simvastatin (40 mg/day) showed no significant effects on dyskinesia, functional impairment or involuntary movement [88].

Neuroprotective mechanisms thought to underlie statins’ role in the prevention of PD are varied. Considering the accepted role of neuroinflammation in PD aetiology, the reduction of neuroinflammation is a common theory. Simvastatin (1.5 µM) has been shown to reduce 6-hydroxydopamine (6-OHDA)-induced TNF-α, IL-6 and cyclooxygenase (COX)-2 up-regulation *in vitro*, and attenuated the up-regulation of caspase-3 via the phosphoinositide 3-kinase (PI3K)/Akt pathway [89]. In cultured rat microglia, high-dose simvastatin (5–20 mM) was found to inhibit LPS-induced inflammatory mediators TNF-α, nitric oxide and superoxide [90]. Statin-induced reductions in neuroinflammatory markers has also been identified *in vivo*, with both simvastatin (30 mg/kg/day, 14 days) and atorvastatin (20 mg/kg/day, 14 days) found to reduce 6-OHDA-induced TNF-α and IL-6 elevations, in addition to reduced oxidative stress markers, including nitrite levels, lipid peroxidation, and restoration of reduced glutathione [91].

Furthermore, animal studies have shown simvastatin (10 mg/kg/day, 21 days) to protect against 6-OHDA-induced loss of *N*-methyl-d-aspartate (NMDA) receptors in rats [92]. Both simvastatin (1 mg/kg/day) and pravastatin (80 mg/kg/day) were also found to attenuate 1-methyl-4-phenyl-1,2,3,6-tetrahydropyridine (MPTP)-induced dopaminergic neuronal loss through inhibition of p21(Ras)-induced NF-κB, though simvastatin appeared to do so more effectively [93]. A number of studies have also observed statin-induced improvements in behavioural activity and motor function in a number of PD models *in vivo* which correlates with protection against induced neuronal damage [88,91,92,93]. Despite encouraging evidence from both cell and animal studies, the lack of prospective and clinical studies into statins’ effects on PD limits our understanding of these drugs in this condition, and hence any conclusions regarding their therapeutic potential.

### 4.4. Multiple Sclerosis

MS is a chronic inflammatory disease of the nervous system, whereby T-cell-mediated responses are associated with the destruction of myelin sheaths, which can ultimately result in axonal damage and neurological deficit [94]. In general, statins have been considered largely beneficial in pathologies associated with demyelination, particularly MS. Although phase II clinical trials of simvastatin treatment in MS patients have recently been successfully completed [95], *in vitro* and *in vivo* evidence surrounding the effects of statins on nerve conduction and remyelination is largely conflicting. It is believed that much of this contradicting data stems from differing experimental designs, including time-dependent responses, and serum conditioning with either foetal bovine serum supplementation or exogenous cholesterol [96,97].

Several statins, including atorvastatin, lovastatin and simvastatin, have been associated with enhanced differentiation of oligodendrocyte progenitor cells (OPCs), the depletion of which exhausts remyelination capacity. Atorvastatin pre-treatment (5 mg/kg/day, 7 days) in an animal model of sciatic nerve crush injury was found to up-regulate several remyelination-associated genes, including growth-associated protein-43, myelin basic protein, ciliary neurotrophic factor, and collagen [98]. This was also associated with an increased protection against damage, including reduced structural disruption, inflammation and neurobehavioural changes [98]. Simvastatin (5–10 µM) has also been associated with inducing process extension in OPCs, and enhanced differentiation to the mature OD phenotype. Interestingly, however, this protective effect was found to be time-dependent, with increased simvastatin exposure time associated with process retraction in both OPCs and mature ODs [99].

The enhanced differentiation of OPCs in the presence of statins has raised the question of whether chronic OPC depletion is likely to affect the regenerative capacity of the neuroparenchyma. Conflicting results have been noted in studies which found detrimental effects of statin treatment on remyelination. Whilst simvastatin (2 mg/kg/day) did not impact myelin load or demyelination in healthy mice over a two week administration period, when extended to five weeks rates of demyelination significantly increased [97]. In the same study, simvastatin decreased myelin load during concomitant demyelination and impeded remyelination, which was attributed to inhibition of OPC differentiation. These results were replicated by Klopfliesch and colleagues who further identified that simvastatin (5 µM) impaired the p21Ras/ p38 mitogen-activated protein kinase (MAPK) pathway and reduced synthesis of myelin basic protein, myelin proteolipid protein and 2',3'-cyclic nucleotide 3'-phosphodiesterase (CNP) *in vitro* [100]. Simvastatin (5–10 µM)-induced OPC process extension and maturation can be mimicked through ROCK inhibition, and either is partially or fully reversed with isoprenoid metabolites, depending on simvastatin exposure time [99]. Given that the vast majority of cholesterol acquisition in the CNS is through glial synthesis or neuronal reutilisation, with little to no reliance on systemic cholesterol pools, cholesterol availability in ODs is a rate-limiting step for successful myelination [101].

In addition to direct effects on ODs, statins’ effects on neuroinflammation and immunomodulation have also been implicated as possible contributing mechanisms in MS. Lovastatin (2 mg/kg/day) has been found to ameliorate clinical symptoms associated with experimental autoimmune encephalomyelitis (EAE), an animal model of human MS, as well as reduce neuroinflammatory mediators such as iNOS, TNF-α and interferon (IFN)-γ [102,103]. Similarly, atorvastatin (10 mg/kg/day) has also been shown to improve clinical symptoms of EAE, which has been attributed to reduced RhoA geranylgeranylation, impaired T cell responses and altered T helper (Th)1/Th2 inflammatory ratios [104]. Statins are also noted to modulate T cell immunity, a factor which plays a crucial role in autoimmune neuroinflammation. Statins have been found to affect T cell response through the inhibition of Th1 differentiation and migration across the BBB [105,106]. In the presence of statins, myelin-reactive CD4^+^ T cells exhibit reduced TNF-α and IFN-γ secretion, and instead secrete protective Th2 cytokines, such as IL-4 [105,106].

It is thought that the negative effects of statins which have been observed *in vitro* may be due to depletion of the isoprenoid GGPP, ordinarily responsible for activation of RhoA signalling [96,99,103]. RhoA-mediated inhibition of ROCK synthesis due to statin treatment induces MAPK, and peroxisome proliferator-activated receptor (PPAR)-γ activators [96]. The activation of PPAR-γ induces phosphatase and tensin homolog (PTEN), which ultimately inhibits OPC proliferation through inducing cell cycle inhibitory proteins [96,107]. The inhibition of Ras and Rho signalling by simvastatin (5 µM) was found to hamper myelin and OD process formation *in vitro* [100]. The reasons underlying discrepancies between cell and animal models of MS are not yet fully understood, however the extent of statin penetration and additional compensatory mechanisms within the whole brain compared to *in vitro* models may be possible explanations. Ultimately, even though underlying mechanisms currently remain elusive, the successful completion of simvastatin in phase II testing as a treatment for MS indicates that this compound may have some benefit in demyelinating conditions [95]. Further information from this trial will be necessary to properly evaluate simvastatin’s role in myelination, with a view to clarifying if this effect is a class or compound-specific action.

### 4.5. Neurofibromatosis Type I

Neurofibromatosis type I (NF-1; formally known as von Recklinghausen disease) is an autosomal dominant disorder associated with learning disabilities and attention deficits, amongst other manifestations. Cognitive dysfunction is the most common neurological complication of NF-1 during childhood [108]. Lovastatin (10 mg/kg/day) was shown to normalise Ras activity, reverse learning and attention deficits and rescue long-term potentiation deficits in a mouse model of NF-1 [109]. Despite a phase I study suggesting that lovastatin (20–40 mg/day, 3 months) treatment in 10–17 year-old children with NF-1 may have potential benefits on cognitive parameters [110], a recent randomised controlled trial found no effect of simvastatin (10–40 mg/day, 12 months) on cognitive deficits or behavioural outcomes in children aged 8–16 with NF-1 [111]. Mechanistic studies as to whether compound-specific effects are seen in NF-1 may be warranted before further clinical evaluation is conducted.

## 5. Statins and Neurological Disease

In addition to effects on cognition, statins have been identified as possible preventative and/or treatment options in a number of neurological conditions, including stroke, epilepsy, depression, cancer and brain and spinal cord injury (see summary of mechanisms and evidence in Table 3). Similar to studies which explore the effects of statins on neurocognitive disorders, there is a lack of information surrounding the molecular mechanism of action of statins in the majority of the neurological disorders discussed in this review. Again, due to the limited data, whether the mechanisms which have been identified thus far are broadly applicable to all statins or solely to the statin tested is often unclear and requires further well-designed studies to be conducted.

**Table 3:** Summary of statins’ effects on neurological disorders.

Disorder	Possible Statin-Induced Mechanisms	Strength of Evidence	Overall Consensus
Stroke	Modulation of eNOS; ↓ nitric oxide; ↓ ROS; ↓ MMPs.	Many *in vitro* and *in vivo* studies. Supported by meta-analyses and well-designed randomised controlled trials.	Statins reduce incidence of ischemic and haemorrhagic stroke, likely through antioxidant effects.
Epilepsy	Lipid raft disruption; ROCK inhibition; ↑ PI3K pathway activity.	Limited *in vitro* and *in vivo* studies.	Very different excitoprotective properties between statins. More studies are required.
Depression	Modulation of NMDA receptor activity; ↓ nitric oxide.	Mainly epidemiological studies. Recent meta-analysis suggested statins reduce risk of depression. Limited mechanism-based studies.	Whether the observed effects from qualitative studies are statin-induced, due to decreased cholesterol, or due to an improved quality of life, or a combination is unclear.
Psychiatric disorders	Unknown.	Limited observational studies.	Causality is unclear. If prevalence is affected by statins, it is thought to be rare and only in predisposed patients.
CNS cancers	↑ caspase-3-mediated apoptosis; cell-cycle arrest; ↓ ERK1/2; ↓ Akt activity.	Limited *in vitro* and *in vivo* studies. Retrospective studies suggest no link between statin use and cancer incidence.	Further *in vivo* studies should be used to clarify statins’ effects. Directed epidemiological studies would also prove useful.
Brain and spinal cord injury	↓ apoptosis; ↓ inflammation; ↓ RhoA/ROCK activity; ↓axonal degradation; ↓ myelin degradation.	Numerous *in vivo* studies.	Statins appear to exert beneficial effects *in vivo* if initiated immediately post-TBI/SCI. Due to some conflicting data, further well-designed studies are required before clinical application can be assessed.

### 5.1. Stroke

In addition to their well-established cardiovascular benefits, randomised controlled trials and meta-analyses have found statin use to be associated with a reduced incidence of ischemic and haemorrhagic stroke [3,4], and improved outcomes neurological outcomes and prognosis acutely following stroke across a number of studies [112,113]. Additionally, recent studies have also identified that statin withdrawal is associated with worsened post-stroke survival [114], and that statin initiation within 24 h of thrombolysis may also improve both short- and long-term outcomes [115]. Although the relationship between stroke and cholesterol levels remains unclear, statins’ systemic effects on the vascular system are thought to underpin much of their effects in stroke, and include antithrombotic effects, anti-inflammatory effects, improved endothelial function, and the stabilising of atherosclerotic plaques.

Several lines of evidence suggest that the modulation of endothelial nitric oxide synthase (eNOS) and reduction of nitric oxide production by statins acts as a primary neuroprotective mechanism against stroke through the improvement of cerebral blood flow around cerebral penumbra [77,116]. In a mouse model of stroke, the protective effects of simvastatin (20 mg/kg/day, 14 days) on infarct size, cerebral blood flow and neurological function were eliminated following eNOS-knockout [117]. Statin-induced increases in eNOS have been attributed to GGPP inhibition [116], subsequent reduction in RhoA and Rac1 expression and the stabilisation of eNOS mRNA [118].

Additionally, several studies have implicated statin-induced reduction in ROS and matrix metalloproteinases (MMPs) in exerting neuroprotective benefits in stroke. The release of MMPs by astrocytes and microglia are associated with neuroinflammation and BBB disruption [119,120]. Several lines of evidence suggest that statin-induced reductions in MMPs may play a role in the apparent immunomodulatory effects of statins. Atorvastatin has been shown to reduce recombination human tissue plasminogen activator (rht-PA)-induced MMP up-regulation in the rat brain, and reduced MMP-associated blood–brain barrier permeability increases [121]. In cortical astrocytes, simvastatin (1–10 µM) significantly reduced rht-PA-induced MMP-9 dysregulation through modulation of the Rho signalling pathway [122]. Similarly, ROS are thought to contribute to ischemia through direct intracellular damage to proteins, lipids and nucleic acids. In rats, atorvastatin pre-treatment (10 mg/kg/day, 3 doses) prior to middle cerebral artery occlusion significantly reduced infarct volume, which coincided with significantly reduced penumbral nicotinamide adenine dinucleotide phosphate (NADPH) oxidase activity and superoxide levels [123]. Similarly, rosuvastatin (2 mg/kg/day, 24 h and 28 day) has been shown to reduce NADPH oxidase-dependent superoxide production in cerebrovascular arteries of insulin-resistant Zucker obese rats [124]. Considering the plethora of data supporting the antioxidant effects of various statins in reducing endothelial dysfunction within the cardiovascular system, it is likely that the observed benefits of statins in cerebrovascular ischemia may also be mediation through reduced ROS activity.

### 5.2. Epilepsy

The incidence of developing epilepsy has two predominant peaks across the human lifespan: during childhood, and after age fifty. Whilst the true pathophysiology is largely unknown, it has been suggested that epilepsy which develops later in life may be a result of cerebrovascular disease, brain tumours or AD. In several epidemiological studies, statin users have been associated with a reduced risk of developing epilepsy, a finding which is supported by studies in animals and *in vitro* [125,126,127,128]. In a case-control study, a dose-dependent effect between statin and seizure risk was observed, with every 1 gram increase in atorvastatin used annually associated with a 5% reduced risk of hospitalisation due to seizure [126].

Although the use of cell culture for modelling seizure mechanisms and epileptogenesis is limited, *in vitro* studies have suggested that statins may exhibit excitoprotective properties, though not at equipotent doses. In primary neuronal cultures, simvastatin was found to reduce the association of subunit 1 of NMDA receptors to lipid rafts by 42%, a mechanism which was hypothesised to contribute to simvastatin-induced protection against NMDA-induced neuronal damage [129]. Lipid rafts are distinct, highly dynamic sterol and sphingolipid-rich microenvironments within the cellular membrane and are implicated as platforms for numerous signalling pathways, thus the perturbation of these zones has the potential to affect neuronal signalling. In addition to simvastatin’s effects on lipid rafts, both simvastatin and lovastatin have also been associated with excitoprotection mediated through the inhibition of calcium-dependent calpain activation, ROCK inhibition, the activation of the PI3K pathway, and increased APP cleavage [125].

Whether all statins contribute equally to this observed excitoprotection remains questionable. An earlier study by Zacco and colleagues identified that a number of statins were capable of protecting primary neurons against NMDA-induced cytotoxicity, though neuroprotective potency differed between statins: (rosuvastatin, simvastatin) > (atorvastatin, mevastatin) > pravastatin [130]. In contrast to these *in vitro* findings, a study in mice comparing five commercially available statins identified simvastatin and lovastatin as effective in reducing seizure severity and histopathological signs of excitotoxicity, whilst neither fluvastatin, atorvastatin nor pravastatin showed any significant benefits in ameliorating seizure-related sequelae [131]. It should be noted however that the protective effects may only be seen at high doses, with a recent rat model of epileptogenesis identifying that a dose of 10 mg/kg/day of either atorvastatin or simvastatin significantly reduced the development of absence seizures, although this dose of pravastatin was ineffective at reducing seizure incidence. Increasing the pravastatin daily dose to 30 mg/kg/day resulted in a significant reduction in number of seizures [132]. Due to the limited data available thus far, further studies are required to evaluate the clinical implications of these findings.

### 5.3. Depression

Similarly to other neurological disorders, there remains conflict across the literature with regards to statins’ effects in depression. Epidemiological evidence has suggested a possible role for statins in the reduction of depression and depression-like symptoms [133,134,135,136,137,138], with a recent meta-analysis by Parsaik and colleagues concluding that statin use was associated with a lower risk for depression (adjusted odds ratio, 0.68; 95% confidence interval, 0.52–0.89) [1]. In addition to all-cause depression, statins have also been linked to a reduced risk of post-stroke depression [136] and augment the increased risk of depression associated with hyperlipidaemia [139]. However, a number of studies have found no significant relationship between statin use and risk of depression or depression-like symptoms [140,141,142], whilst one study found that statin use was associated with increased depression prevalence [43]. Thus, whether the apparent protective effect of statins against depression is a true pharmacological effect, or a result of other factors, such as improved cardiovascular health or increased health consciousness following statin treatment, remains unclear.

This uncertainty is compounded by a lack of mechanism-based studies which explore the anti-depressant effects of statins in animal models. In rats exposed to chronic mild stress, simvastatin (5–10 mg/kg/day, 14 days) reversed some stress-induced behavioural changes comparable to imipramine, a tricyclic antidepressant [143]. Similarly, atorvastatin (0.1–10 mg/kg, single dose) has been shown to exhibit acute antidepressant-like activity in mice, with modulation of NMDA receptor activity and nitric oxide inhibition identified as possible mechanisms [144]. Further well-designed animal studies which explore the relationship between statin use, hypercholesterolaemia, anxiety and depression are warranted.

### 5.4. Psychiatric Disorders

Studies designed to determine statins’ effects on specific neuropsychiatric reactions are limited and have yielded conflicting results. Statin use was not associated with any alterations in risk of schizophrenia, schizoaffective disorders, psychosis, major depression, or bipolar disorder compared to non-users in an observational, propensity score-matched cohort study [145]. In contrast, one study found that statin use was associated with reduced risk of anxiety and hostility [134]. Due to the limited reports of negative psychiatric events and a lack of causality, it is largely thought that psychiatric events associated with statins are rare, perhaps occurring only in predisposed patients.

### 5.5. CNS Cancers

The effect of statins on both cancer incidence and mortality remains unclear, with evidence for both reduced and increased cancer-related mortality associated with statin use [146,147]. Although large scale meta-analyses have suggested that statins do not have significant effects on cancer incidence [51,148,149], evidence from both cell and animal studies has suggested a possible role for statins in the treatment of cancers. Of these studies, however, only a limited number have been conducted using neurological models.

An early phase I study by Thibault and colleagues determined the effects of lovastatin in 88 patients, of which 24 patients had tumours of the primary central nervous system. Whilst this study observed that lovastatin (25 mg/kg daily for 7 consecutive days) was well-tolerated in both healthy and cancer patients, effects on cancer progression were not sought [150]. Similarly, a subsequent phase I/II trial using lovastatin (35 mg/kg) in patients with anaplastic astrocytoma and glioblastoma multiforme, no CNS toxicity associated with treatment was found, however no improvement in tumour response was observed [151]. Of the remaining studies which report on cancer risk associated with statin use, the majority are not designed to determine effects on cancer as a primary endpoint, thus it is difficult to ascertain the true clinical effect of statins in cancer, particularly those of a CNS origin.

As such, the majority of data thus far stems from cell and animal models. Several cancer models have been investigated, with statins appearing to exert beneficial anti-tumourogenic effects in animal models of glioma (lovastatin) [152] and neuroblastoma (mevinolin, lovastatin) [153,154]. In an *in vitro* model, lovastatin was found to reduce the invasiveness of human glioma cells [155]. Several statins (lovastatin, mevastatin, fluvastatin and simvastatin) have also been found to increase caspase-3 mediated apoptosis and decrease extracellular-signal-regulated kinases (ERK) 1/2 and Akt, also known as protein kinase B, in C6 glioma cells through GGPP-dependent mechanisms [156,157]. Similarly, lovastatin-induced apoptosis in SH-SY5Y neuroblastoma cells is mediated through GGPP-dependent mechanisms [158]. *In vivo*, both simvastatin and lovastatin have been shown to reduce malignant rat gliomas and murine neuroblastoma growth respectively, with simvastatin’s effects attributed to growth arrest and induction of apoptosis [152,153]. Ultimately, however, until further epidemiological studies and clinical trials are conducted, the true effect of statins on incidence of CNS cancer and tumour growth remains unclear.

### 5.6. Brain and Spinal Cord Injury

Statins, particularly atorvastatin and simvastatin, have been widely studied *in vivo* for their effects in TBI and spinal cord injury (SCI). On the whole, data thus far suggests a positive, neuroprotective effect induced by statins across both models.

Atorvastatin has been identified across numerous studies as exerting beneficial effects against the neurological sequelae associated with SCI. Atorvastatin-treated rats (5 mg/kg, 2 h post-injury) have shown significant improvement in locomotor activity compared to control rats four weeks post-SCI in rats, which was attributed to reductions in early apoptosis at the injury site [159]. Similar studies in rats have identified additional mechanisms through which atorvastatin may exert its neuroprotective effects in SCI, including reduced blood-spinal cord barrier dysfunction through reduced RhoA/ROCK activity, reduced infiltration and expression of TNF-α, IL-1β and iNOS at the site of injury, reduced axonal degradation, myelin degradation, gliosis and neuronal death [160,161].

Likewise, studies in rats using simvastatin [162,163] and rosuvastatin [164] have observed similar results. Simvastatin (5–10 mg/kg) treatment post-SCI was associated with improved locomotor activity, normalisation of magnetic resonance imaging, increased glial cell-derived neurotrophic factor (GDNF), BDNF, and VEGF expression, and mobilisation of bone marrow stromal cells [162,163]. Rosuvastatin administration (20 mg/kg) immediately post-spinal cord injury in rats reduced elevations in TNF-α release, myeloperoxidase activity, nitric oxide levels and caspase-3 activity from caudal spinal cord tissue [164].

Whilst animal studies thus far have largely supported a beneficial role for statins in improving neurological outcomes following TBI or SCI, not all studies have found neuroprotective benefit following statin treatment in SCI [165]. As such, further evaluation of these compounds is required before the translational value of these data can be accurately assessed.

## 6. Conclusions

Whilst research into understanding statins’ CNS effects has been extensive in recent years, there is still a distinct lack of mechanistic supportive evidence to justify the use of these compounds in the prevention or treatment of neurological disorders. The available mechanistic evidence supports a possible beneficial role of statin treatment in some conditions, such as the prevention of dementia and MS treatment, suggesting that the high concerns over statins’ neurological effects may be largely unwarranted. While it is apparent that the structural differences between statin compounds contribute to their vastly different pharmacokinetic parameters, how this translates into pleiotropic differences between statins is less widely acknowledged. In the CNS in particular, an improved understanding as to the precise mechanistic differences between statins is needed so that therapeutic decision making may be better informed. Until such time that more comparative evidence is available, it would be prudent for clinicians and researchers to consider the evidence for individual statins in the CNS, as opposed to assuming a class action. Additionally, more evidence is required before any statin therapy can be recommended clinically in the treatment or prevention of these neurological conditions.

## Abbreviations

HMG-CoA: 3-hydroxy-3-methylglutaryl coenzyme A
6-OHDA: 6-hydroxydopamine
AD: Alzheimer’s disease
Aβ: amyloid beta peptide
APP: amyloid precursor protein
BBB: blood-brain barrier
BDNF: brain-derived neurotrophic factor
CNS: central nervous system
CSF: cerebrospinal fluid
CYP: cytochrome P450
eNOS: endothelial nitric oxide synthase
EAE: experimental autoimmune encephalomyelitis
ERK: extracellular-signal-regulated kinase
FPP: farnesylpyrophosphate
GGPP: geranylgeranylpyrophosphate
iNOS: inducible nitric oxide synthase
IFN: interferon
IL: interleukin
LPS: lipopolysaccharide
LDL: low-density lipoprotein
MMP: matrix metalloproteinase
MAPK: mitogen-activated protein kinase
MS: multiple sclerosis
NMDA: *N*-methyl-d-aspartate
NADPH: nicotinamide adenine dinucleotide phosphate
NF-1: neurofibromatosis type 1
OD: oligodendrocyte
OPC: oligodendrocyte progenitor cell
PD: Parkinson’s disease
PPAR: peroxisome proliferator-activated receptor
PK: pharmacokinetic
PI3K: phosphatidylinositol-3-kinase
ROS: reactive oxygen species
rht-PA: recombination human tissue plasminogen activator
ROCK: Rho-associated coiled-coil kinase1/2
sAPP: soluble APP
SCI: spinal cord injury
Th: T helper
TBI: traumatic brain injury
TNF: tumour necrosis factor
VEGF: vascular endothelial growth factor
